# The prostaglandin H2 analog U-46619 improves the differentiation efficiency of human induced pluripotent stem cells into endothelial cells by activating both p38MAPK and ERK1/2 signaling pathways

**DOI:** 10.1186/s13287-018-1061-4

**Published:** 2018-11-15

**Authors:** Liping Su, Xiaocen Kong, Szeyun Lim, Szejie Loo, Shihua Tan, Kiankeong Poh, James Dutton, Colin Stewart, Stuart Cook, Xiaofei Su, Jianhua Ma, Jianyi Zhang, Lei Ye

**Affiliations:** 1 National Heart Research Institute of Singapore, National Heart Centre Singapore, Singapore, 117609 Singapore; 2 Department of Endocrinology, Nanjing First Hospital, Nanjing Medical University, 68 Changle Road, Nanjing, 210006 China; 3 Department of Cardiology, National University Health System Singapore and Yong Loo Lin School of Medicine, National University of Singapore, Singapore, Singapore; 4 Stem cell Institute, University of Minnesota, Minneapolis, MN USA; 5 Institute of Medical Biology, A*STAR, Singapore, Singapore; 6 Programme in Cardiovascular & Metabolic Disorders, Duke-National University of Singapore, Singapore, Singapore; 7 NHLI, Imperial College, London, UK; 8 Department of Biomedical Engineering, The University of Alabama at Birmingham, Birmingham, AL 35294-2182 USA

**Keywords:** Human-induced pluripotent stem cells, Endothelial differentiation, Signaling pathways

## Abstract

**Background:**

We have shown that the differentiation of human-induced pluripotent stem cells (hiPSCs) into endothelial cells (ECs) is more efficient when performed with a 3-dimensional (3D) scaffold of biomaterial than in monolayers. The current study aims to further increase hiPSC-EC differentiation efficiency by deciphering the signaling pathways in 3D scaffolds.

**Methods and results:**

We modified our 3D protocol by using U-46619 to upregulate both p38 mitogen-activated protein kinase (p38MAPK) and extracellular signal-regulated kinase 1/2 (ERK1/2) signaling, which increased the differentiation efficiency (as measured by CD31 expression) to as high as 89% in two established hiPSC lines. The differentiated cells expressed arteriovenous, but not lymphatic, markers; formed tubular structures and EC lumen in vitro; had significantly shorter population-doubling times than monolayer-differentiated hiPSC-ECs; and restored perfusion and vascularity in a murine hind limb ischemia model. The differentiation efficiency was also > 85% in three hiPSC lines that had been derived from patients with diseases or disease symptoms that have been linked to endothelial dysfunction.

**Conclusions:**

These observations demonstrate that activating both p38MAPK and ERK1/2 signaling pathways with U-46619 improves the efficiency of arteriovenous hiPSC-EC differentiation and produces cells with greater proliferative capacity.

**Electronic supplementary material:**

The online version of this article (10.1186/s13287-018-1061-4) contains supplementary material, which is available to authorized users.

## Background

Vascular endothelial cells (ECs) form a physical barrier between the vessel wall and lumen, are metabolically active, and play a key role in the maintenance of cardiovascular homeostasis [1, 2] by producing molecules that regulate vascular tone, cell adhesion, clotting, and fibrinolysis [2]. Pathophysiological conditions, such as hyperglycemia, hypercholesterolemia, hypertension, and stress, can lead to EC functional abnormalities that have been linked to atherosclerosis, coronary artery disease, diabetes, and hypertension, as well as normal physiological aging [1–4]. However, the availability of primary human ECs for investigations of cell therapy or to serve as an in vitro platform for drug testing and disease modeling is limited. Human-induced pluripotent stem cells (hiPSCs) could relieve this scarcity because they can be differentiated into theoretically unlimited numbers of any type of cell. Since hiPSCs are generated from a patient’s own somatic cells, they carry genetic variations that may contribute to the development of the patient’s disease [5–10].

Traditional protocols for differentiating hiPSCs into ECs (hiPSC-ECs) are performed in two-dimensional (2D) culture systems [11–17], likely because the endothelium is a 2D tissue. However, we have previously shown that hiPSC-EC differentiation can be remarkably efficient when conducted in three-dimensional (3D) fibrin scaffolds [18]; up to 45% of the hiPSCs assumed an EC phenotype, and the phenotype remained stable for up to 4 weeks in vitro. Here, we investigated the pathways involved in hiPSC-EC differentiation to determine whether our protocol could be made even more efficiently by targeting the p38 mitogen-activated protein kinase (p38MAPK) and extracellular signal-regulated protein kinases 1 and 2 (ERK1/2) signaling pathways, which have been shown to contribute independently to the EC differentiation of pluripotent stem cells [19, 20]. Thus, we investigated the temporal dependence of our hiPSC-EC differentiation protocol on these signaling pathways by treating the cells with selective inhibitors of p38MAPK (losmapimod [Losma]) [21] or ERK1/2 (SCH772984 [*SCH*]) [22] during differentiation stages. We found that our enhanced protocol can not only be used to generate ECs from the non-disease hiPSCs, but also cells of patients whose disease or disease symptoms have been linked to endothelial dysfunction, such as type 2 diabetes and atherosclerosis in patients with Hutchinson-Gilford progeria syndrome [23–25], which have not been achieved with high differentiation efficiency.

## Materials and methods

### hiPSC generation

The five hiPSC lines used in this study were reprogrammed from dermal fibroblasts by using non-integrating Sendai virus and the reprogramming factors OCT4, SOX2, KLF4, and C-MYC, as described previously [26]. PCBC16iPS and GRiPS cells were reprogrammed from neonatal human dermal fibroblasts (Lonza, USA) [18]. PG-608iPS cells were derived from a patient of the Coriell Institute for Medical Research (USA) who had Hutchinson-Gilford progeria syndrome. DP1-C9iPS and DP3-C6iPSC cells were reprogrammed from the cells of two patients with type 2 diabetes mellitus (T2DM). The procedures were approved by the ethics committee of Nanjing Hospital, Nanjing, China, and the Centralised Institutional Review Board of Singapore Health Services Pte Ltd., Singapore; informed consent forms were signed by all patients. PCBC16iPSCs were used as representative hiPSCs in all experiments unless stated otherwise. hiPSCs were cultured in a feeder-free system with a 1:1 mixture of E8/mTeSR (STEMCELL Technologies, Canada) and passaged every 4 days with Versene (Thermo Fisher, USA).

### hiPSC-EC differentiation

#### 3D scaffolds

This differentiation protocol was modified from a protocol that has been described previously [18]. Briefly, stage 1 began 2 days before initiating differentiation, when hiPSCs were dissociated into single cells, seeded into a 0.4-mL fibrin scaffold on a 24-well plate, and transferred to 6-well plates. Stage 2 of the protocol was initiated on day 0 by culturing the cell-containing fibrin/thrombin scaffold in EBM2 medium (Lonza, USA) supplemented with B27 without insulin and CHIR99021 (CHIR) with or without U46619 for 24 h. The third stage began on day 1 when the medium was replaced with EBM2 medium supplemented with B27 without insulin, vascular endothelial growth factor-165 (VEGF), transforming growth factor β1 (TGFβ1), and erythropoietin (EPO); the cells were cultured for 48 h, the medium was refreshed on day 3, and the cells were cultured for another 48 h. On day 5, the differentiating hiPSCs were released and cultured in EGM2-MV medium (Lonza, USA) supplemented with B27, VEGF, and SB-431542 (SB). The medium was changed every 2 days, and differentiation efficiency was evaluated on day 11 via fluorescence-activated cell sorting (FACS); cells positive for CD31 expression and for both CD31 and CD144 expression were collected and expanded. For investigations of p38MAPK and ERK1/2 inhibition, the inhibitors (10 μM Losma, an inhibitor of p38MAPK [21], and/or **5** μM *SCH*, an *inhibitor of ERK1/2* [22]) were added to the differentiation medium 30 min before CHIR, VEGF/TGFβ1/EPO, or U46619 treatment was initiated.

#### 2D monolayers

The monolayer culture protocol was identical to the 3D culture protocol with the following exceptions. In stage 1, the dissociated hiPSCs were seeded into 6-well plates and cultured in monolayers, and on day 5, one well of the differentiating hiPSCs was harvested and cultured in a T-25 flask with EGM2-MV medium supplemented with B27, VEGF, and SB. The medium was changed every 2 days, and differentiation efficiency was evaluated on day 11 via FACS.

The EC population doubling time was calculated within 7 days after cell sorting. Briefly, ECs were harvested on day 2 after purification and cultured in 6-well plates (2 × 10^5^ cells/well). The medium was changed every 2 days, and ECs were harvested and counted on day 7.

### Flow cytometry

Flow cytometry analyses were conducted as described previously [18, 27]. Briefly, the differentiated hiPSC-ECs were trypsinized and re-suspended as single cells in glass tubes, incubated with 2% fetal bovine serum (FBS) in phosphate-buffered saline (PBS) containing primary phycoerythrin (PE)- or allophycocyanin (APC)-conjugated anti-human CD31 antibodies (clone WM59, BD Pharmingen, USA), FITC or PE-conjugated anti-human CD144 antibodies (clone 55-7H1, BD Pharmingen, USA), or isotype control antibodies for 30 min at 4 °C. To determine EC type, purified ECs on day 7 were incubated with FITC-conjugated anti-Eph-B4 antibodies, PE-conjugated anti-human CXC chemokine receptor type 4 (CXCR4) antibodies, APC-conjugated anti-human delta-like 4 (DLL4) or anti-human podoplanin antibodies (Miltenyi Biotec, Germany), or isotype control antibodies for 30 min at 4 °C. The cells were washed with 2% FBS/PBS, re-suspended in 0.3 mL 2% FBS/PBS containing 5 μL of propidium iodide (10 μg/mL), and evaluated with a FACS Aria instrument (BD Biosciences, USA).

### Western blot

Phosphorylated and non-phosphorylated p38MAPK and ERK1/2 protein levels were determined by Western blot analysis as described previously [28]. The cell lysate was prepared with PhosphoSafe™ Extraction Reagent (Merck, Germany), and protein concentrations were determined with Bradford reagent (Bio-Rad Laboratories, USA). Proteins were separated, electrophoretically blotted onto nitrocellulose membranes, and washed with 10 mM Tris-HCl buffer (pH 7.6) containing 0.05% Tween-20; then, the membranes were incubated in blocking buffer (5% non-fat dry milk, 10 mM Tris pH 7.5, 100 mM NaCl, 0.1% Tween-20) at room temperature for 3 h and with diluted primary antibodies (glyceraldehyde phosphate dehydrogenase [GAPDH 1:2000, pERK1/2 [Thr202/Tyr204] 1:1000, Santa Cruz Biotech, USA; p-p38MAPK [Thr180/Tyr182] 1:500, ERK1/2 1:1000, and p38MAPK 1:1000, Cell Signaling, USA) at 4 °C overnight. Bound antibodies were detected with HRP-conjugated anti-rabbit IgG (dilution 1:1000 and 1:8000) and visualized with a ChemiDocTM MP Imaging System (Bio-Lab, USA) and Image Lab 5.1 software (Bio-Lab, USA).

### Quantitative RT-PCR

RNA isolation and cDNA synthesis was performed as described previously [26, 29], and PCR thermal cycling was conducted with the following primers: Brachyury, forward: AAAGAGATGATGGAGGAACCCGGA, reverse: AGGATGAGGATTTGCAGGTGGACA; Etv2, forward: GGGCTTGAAGGAGCCAAATTA, reverse: CAGGGATGAGCTTGTACCTTTC; Gata-2, forward: GACGACAACCACCACCTTAT, reverse: AGTCTGGATCCC TTCCTTCT; Tal-1, forward: AAATGGAGCAAAGTGGTAGGT, reverse: GTGACAACTCCAGCCTCTTAC; CD34, forward: TAGCCTGTCACCTGGAAATG, reverse: TGCCTTGATGTCACTTAGGATAG; and CD31, forward: TTGAGACCAGCCTGATGAAACCCT, reverse: TCCGTTTCCTGGGTTCAAGCGATA. Thermal cycling was performed 40 times, and each cycle consisted of enzyme activation at 95 °C for 15 min, denaturation at 95 °C for 30 s, and annealing at 60 °C (for all PCR reactions) for 30 s and extension at 72 °C for 30 s. Endogenous GAPDH (forward: TCGACAGTCAGCCGCATCTTCTTT, reverse ACCAAATCCGTTGACTCCGACCTT) levels were used as an internal control for normalization [26]. Brachyury expression was presented as a percentage of measurements obtained after Activin-A/BMP-4 treatment, and the expression of other genes was presented as a percentage of their expression at day 0.

### Dil-conjugated acetylated low-density lipoprotein uptake and tube formation

Dil-conjugated acetylated low-density lipoprotein (Dil-ace-LDL) uptake and tube formation were evaluated as described previously [18]. For the Dil-ace-LDL uptake assay, hiPSC-ECs were incubated with DAPI overnight (1:1000 dilution) and then in EGM supplemented with 10 μg/mL of Dil-ace-LDL (Life Technologies, USA) at 37 °C for 12 h. For the tube formation assay, cells were seeded in 48-well plates that had been coated with Matrigel (BD Pharmingen, USA) and incubated at 37 °C for 24 h. Numbers of node, junction, and mesh per low magnification (× 4) were quantified using angiogenesis analyzer of ImageJ. For tube formation in 3D, 2 × 10^4^ hiPSC-ECs were seeded into 3D fibrin-thrombin scaffolds composed of 50 μL of 25 mg/mL fibrinogen and 50 μL of 20 U/mL thrombin and cultured in EGM supplemented with a × 100 dilution of B27, 100 ng/mL VEGF, SB, and 100 U/mL aprotinin.

### Murine hind limb ischemia model and treatment

The experimental protocol and animal maintenance procedures were approved by the Institutional Animal Care and Use Committee and performed in accordance with the Animal Use Guidelines of Singapore Health Services Pte Ltd. Eight-week-old NOD-SCID mice (InVivos, Singapore) were anesthetized with intraperitoneal injections of ketamine (100 mg/kg) and xylazine (2.5 mg/kg). The right limb was shaved and disinfected with betadine and 70% alcohol; then, the femoral artery of the right hind limb was exposed and freed from the inguinal ligament via a longitudinal incision extending to a point just proximal to the patella. The artery and all branches from the inguinal ligament to the point where it bifurcates into the popliteal and saphenous arteries were closed with 6-0 polypropylene sutures; then, the wound was closed, and the animals were allowed to recover. Ketoprofen (2.5 mg/kg, subcutaneous) was administered for pain control and Baytril (15 mg/kg, intramuscular) to prevent infection for at least 3 days after the surgical procedure. Animals were randomly assigned to treatment with 1.5 × 10^6^ hiPSC-ECs in 0.2 mL EBM (i.e., the hiPSC-EC group, *n* = 8) or with 0.2 mL EBM (i.e., the basal medium [BM] group, *n* = 9). The hiPSC-ECs had been differentiated from the PCBC16 cell line, and the treatments were administered 3 days after hind limb ischemia (HLI) induction via four intramuscular injections into the center of the ligated area and the surrounding region along the femoral artery.

### Laser Doppler imaging

Mice were anesthetized with intraperitoneal injections of ketamine (100 mg/kg) and xylazine (2.5 mg/kg), their hind limbs were shaved, and laser Doppler imaging was performed with a PeriScan PIM 3 System (Perimed, Sweden). Measurements in the ischemic (right) limb were normalized to measurements in the non-ischemic (left) limb and expressed as a percentage.

### Immunohistochemistry

For characterization of hiPSC-ECs in vitro, cells were fixed with 4% paraformaldehyde for 20 min at room temperature and then blocked with UltraV block (Fisher Scientific, USA) for 7 min. Primary antibodies (monoclonal anti-CD31 and mouse anti-CD144 [BD Pharmingen, USA]; 1:100 concentration) were added to the UltraV block buffer and incubated overnight at 4 °C; then, the cells were incubated with PE-conjugated goat anti-mouse IgG secondary antibodies in PBS for 1 h at room temperature, labeled with 4′,6-diamidino-2-phenylindole (DAPI), washed, and viewed under a fluorescence microscope (Olympus, Japan).

To determine the neovascularization in ischemic limb, mice limb muscles were collected, frozen, and cut into 8-μm-thick sections; then, the sections were stained for CD31 expression (rabbit anti-CD31 [Abcam, USA] which targets both human and mouse ECs and goat anti-rabbit IgG conjugated with FITC [Thermo Fisher Scientific, USA]) to evaluate total vessel density, for smooth muscle actin (SMA) expression (Cy3-conjugated mouse anti-SMA antibodies [Sigma-Aldrich] which targets both human and mouse SMCs) to evaluate arteriole density. Vascular structures that were positive for CD31 expression (i.e., FITC fluorescence) and for both CD31 and SMA expression (i.e., simultaneous FITC and Cy3 fluorescence) were counted for all animals in both groups, three to four slides per animal, six to eight fields per slide.

To identify transplanted hiPSC-ECs, a primary antibody specifically against human CD31 (hCD31, mouse anti-human CD31-Biotin) was used and visualized by mouse anti-Biotin-VioBright 515 (both from Miltenyi Biotec, Germany). Fluorescence images were taken with an Olympus IX71 fluorescence microscope.

### Statistics

Data are presented as mean ± standard deviation (SD). Comparisons among groups were analyzed for significance via one-way analysis of variance (ANOVA) with the Tukey correction. Analyses were performed with SPSS software. A value of *p* < 0.05 was considered significant.

## Results

### CHIR99021 dose-dependently promotes the mesodermal specification of hiPSCs

**Fig. 1:**
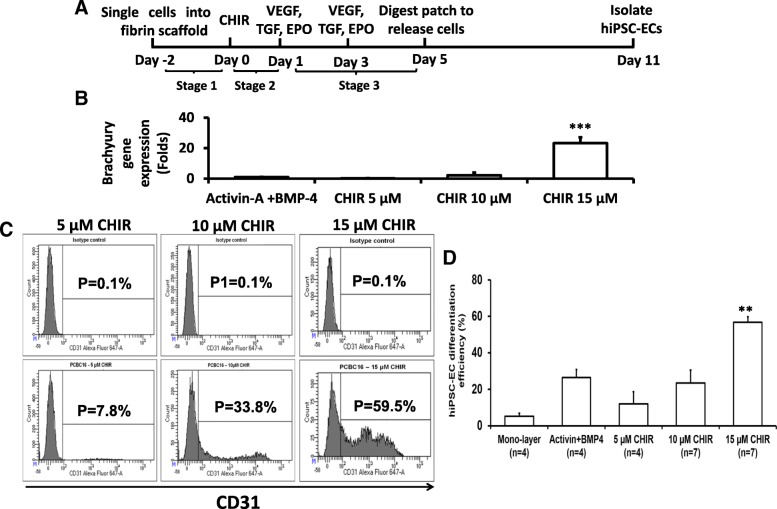
**a** A schematic diagram of the hiPSC-EC differentiation protocol with CHIR99021 (CHIR). **b** Brachyury gene expression as a function of CHIR concentration. **c** Representative flow cytometry analysis of CD31 when hiPSC-EC differentiation was conducted with 5, 10, or 15 μM CHIR. **d** hiPSC-EC differentiation was calculated as the percentage of CD31^+^ cells for cells differentiated under the indicated conditions. (**p < 0.01 vs any other treatment)

The 3D differentiation protocol consists of three stages (Fig. 1a). In stage 1, the cells were seeded into the 3D scaffold and maintained under standard hiPSC culture conditions for 2 days (i.e., from day − 2 to day 0). Differentiation begins in stage 2 (day 0 to day 1) when the cells are directed toward an intermediate, mesodermal lineage; then, the final hiPSC-EC phenotype is induced during stage 3 (day 1 to day 5) by exposing the cells to VEGF, TGFβ1, and EPO. In our previous report, stage 2 was initiated by culturing the cells with 50 ng/mL Activin-A and 25 ng/mL BMP-4 [18]; however, mesodermal commitment can also be induced with the glycogen synthase kinase 3α/β inhibitor, CHIR [30], so we compared expression of the early mesodermal marker, Brachyury, in hiPSCs cultured with varying concentrations of CHIR. Brachyury mRNA levels increased logarithmically as CHIR concentrations were raised from 5 to 15 μM in 5 μM increments, and after 24 h of differentiation, measurements were ~ 23-fold greater with 15 μM CHIR than when differentiation was initiated with Activin-A/BMP-4 (Fig. 1b). Furthermore, upon completion of the entire hiPSC-EC differentiation protocol, flow cytometry assessments of CD31 expression (Fig. 1c) indicated that the efficiency of differentiation increased from 10 to 58% over the same range of CHIR concentrations and was ~ 2-fold greater with 15 μM CHIR than with Activin-A/BMP-4 (Fig. 1d, *p* < 0.01). Thus, the mesodermal commitment was initiated by culturing the hiPSCs with 15 μM CHIR, rather than Activin-A/BMP4, for all subsequent experiments.

### hiPSC-EC differentiation declines when p38MAPK signaling or ERK1/2 signaling is inhibited

Because the p38MAPK and MEK/ERK signaling pathways contribute independently to the EC differentiation of pluripotent stem cells [19, 20], we investigated the temporal dependence of our hiPSC-EC differentiation protocol on these signaling pathways by treating the cells with selective inhibitors of p38MAPK (10 μM Losma) or ERK1/2 (**5** μM SCH) during each of the three stages. Western blot analyses confirmed that treatment with Losma or SCH did not alter ERK1/2 or p38MAPK signaling, respectively (Additional file 1: Figure S1).

**Fig. 2:**
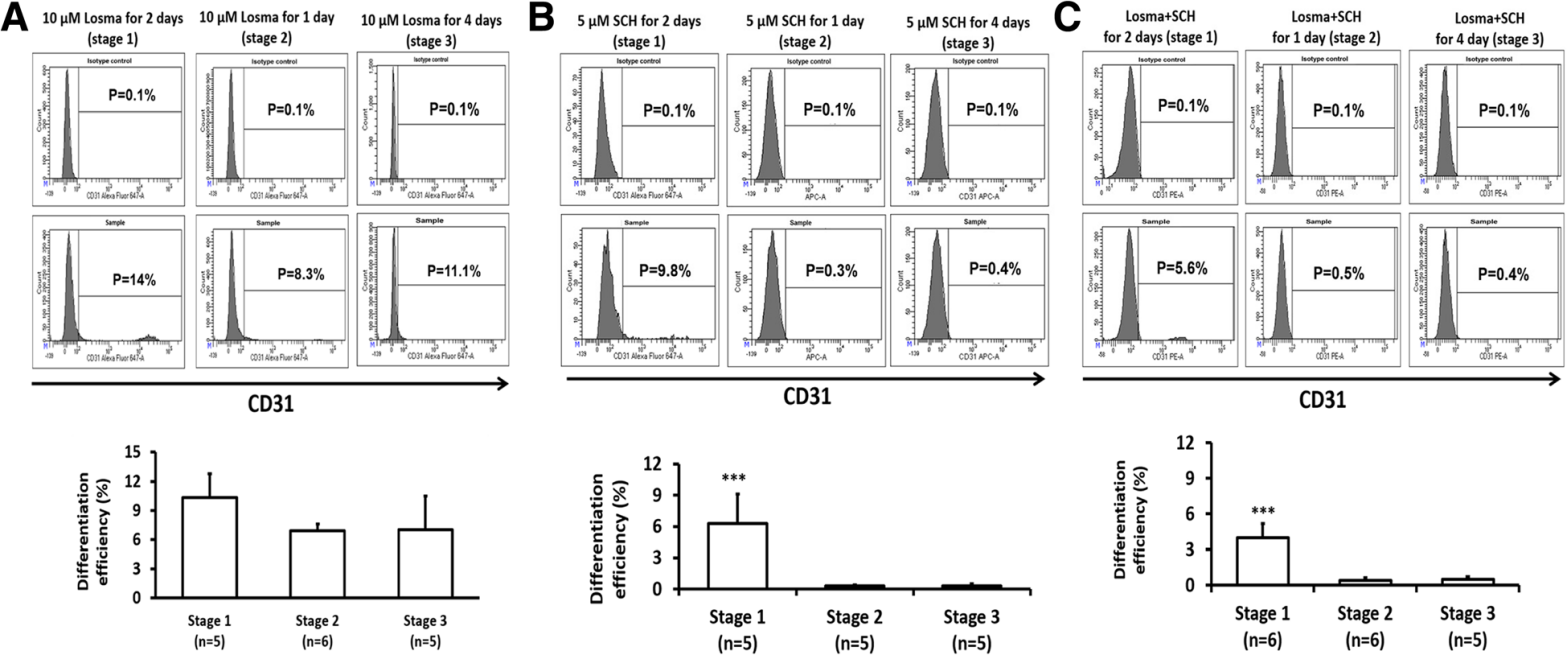
**a** Representative flow cytometry results and hiPSC-EC differentiation efficiency when 10 μM of the p38MAPK inhibitor losmapimod (Losma) was supplemented in differentiation stage 1, 2, or 3. **b**. Representative flow cytometry results and hiPSC-EC differentiation efficiency when 5 μM of the ERK1/2 inhibitor SCH was supplemented in differentiation stage 1, 2, or 3. **c** Representative flow cytometry results and mean hiPSC-EC differentiation efficiency when both Losma and SCH were supplemented in differentiation stage 1, 2, or 3. Assessments of CD31 levels were compared with isotype controls. (***p < 0.001 vs any other treatment)

Flow cytometry analyses of CD31 expression indicated that the differentiation efficiency declined to 6–10% with Losma treatment in all three stages (Fig. 2a) and with SCH (Fig. 2b) treatment in stage 1. Differentiation was also substantially impaired by combined Losma and SCH treatment in stage 1; however, when SCH was added either alone or in combination with Losma during stage 2 or stage 3, differentiation was completely blocked. Furthermore, qRT-PCR analyses indicated that when added in stage 2 (Additional file 1: Figure S2A-F), SCH, but not Losma, reduced Brachyury mRNA levels, as well as the expression of the hematopoietic stem cell marker CD34. Thus, ERK1/2 signaling, but not p38MAPK signaling, appears to have a key role in the mesodermal specification of hiPSCs. Both treatments during stage 2 led to declines in CD31 mRNA levels, and expression of the endothelial transcription factors Evt2, Gata-2, and Tal-1 were either reduced (Evt2, Gata-2) or delayed (Tal-1) in Losma-treated cells; the expression of all three factors declined in response to SCH treatment.

When SCH was added during stage 3, the expression of all three endothelial transcription factors, as well as CD34 and CD31, declined (Additional file 1: Figure S2G-K); whereas treatment with Losma in stage 3 led to declines in Gata-2, CD34, and CD31 mRNA, but Etv2 and Tal-1 expression were largely unchanged. Collectively, these observations suggest that ERK1/2 signaling is crucial for hiPSC-EC differentiation, particularly during stages 2 and 3 of our protocol, whereas the p38MAPK signaling pathway may play in more of an auxiliary role.

### U-46619 improved hiPSC-EC differentiation efficiency by activating both p38MAPK and ERK1/2 signaling

**Fig. 3:**
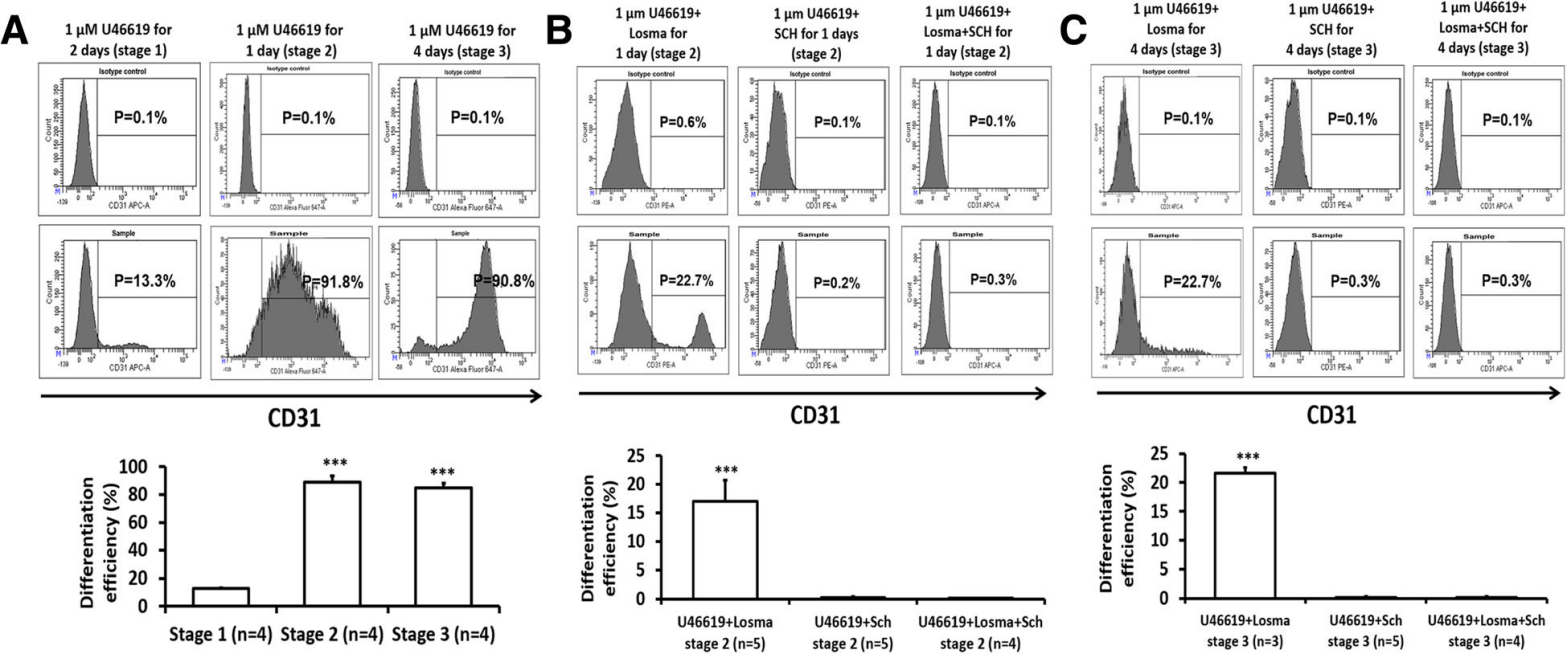
**a** Representative flow cytometry results and hiPSC-EC differentiation efficiency when 1 μM U46619 was supplemented in differentiation stage 1, 2, or 3 (***p < 0.001 vs stage 1). Representative flow cytometry results and hiPSC-EC differentiation efficiency when U46619 was co-supplemented with Losma, SCH, or both Losma and SCH during differentiation stage 2 (**b**) or 3 (**c**) (***p < 0.001 vs any other treatment). Assessments of CD31 levels were compared with isotype controls

Because our observations indicated that both the p38MAPK and MEK/ERK pathways contribute to hiPSC-EC differentiation, we evaluated whether the efficiency of our hiPSC-EC differentiation protocol could be improved by supplementing the medium with the prostaglandin H2 analog U-46619, which has been shown to activate p38MAPK and ERK1/2 signaling [31, 32]. Experiments were conducted with hiPSCs from the PCBC16iPS, which were reprogrammed from neonatal human dermal fibroblasts and have been used extensively in another investigation [18]. U-46619 did not improve differentiation efficiency when added during the first stage of our protocol; however, when 1 μM U-46619 was added during stage 2 or stage 3, > 85% (stage 2 89.1 ± 4.3%, stage 3 85 ± 3.2%) of the differentiated cells expressed CD31 (Fig. 3a). Higher U-46619 concentration (5 μM) was less effective at promoting hiPSC-EC differentiation; < 40% of the differentiated cells expressed CD31 when 5 μM U-46619 was added to the medium during protocol stage 2 or stage 3 (Additional file 1: Figure S3). Differentiation also declined to < 25% when U-46619 treatment was combined with Losma and was completely blocked when U-46619 was combined with SCH or both SCH and Losma in stage 2 or 3 (Fig. 3b, c). Western blots confirmed that Losma specifically inhibited U-46619-induced p38MAPK activity, that SCH specifically inhibited U-46619-induced ERK1/2 activity, and that the combination of Losma and SCH inhibited the U-46619-induced activity of both pathways (Additional file 1: Figure S4). Furthermore, qRT-PCR analyses indicated that when added in stage 2 (Additional file 1: Figure S5A-F) or stage 3 (Additional file 1: Figure S5G-J), U-46619 enhanced or prolonged Etv2, Gata-2, Tal-1, CD34, and CD31 gene expression but not in the presence of SCH alone or combined SCH/Losma treatment. Losma impeded the expression of all five markers when added to U-46619-treated cells in stage 2; however, when added in stage 3, Etv2 expression in U-46619-treated cells was prolonged, while the expression of Gata-2, Tal-1, CD34, and CD31 gene expression was largely unchanged (Gata-2, Tal-1) or only moderately reduced (CD34, CD31).

**Fig. 4:**
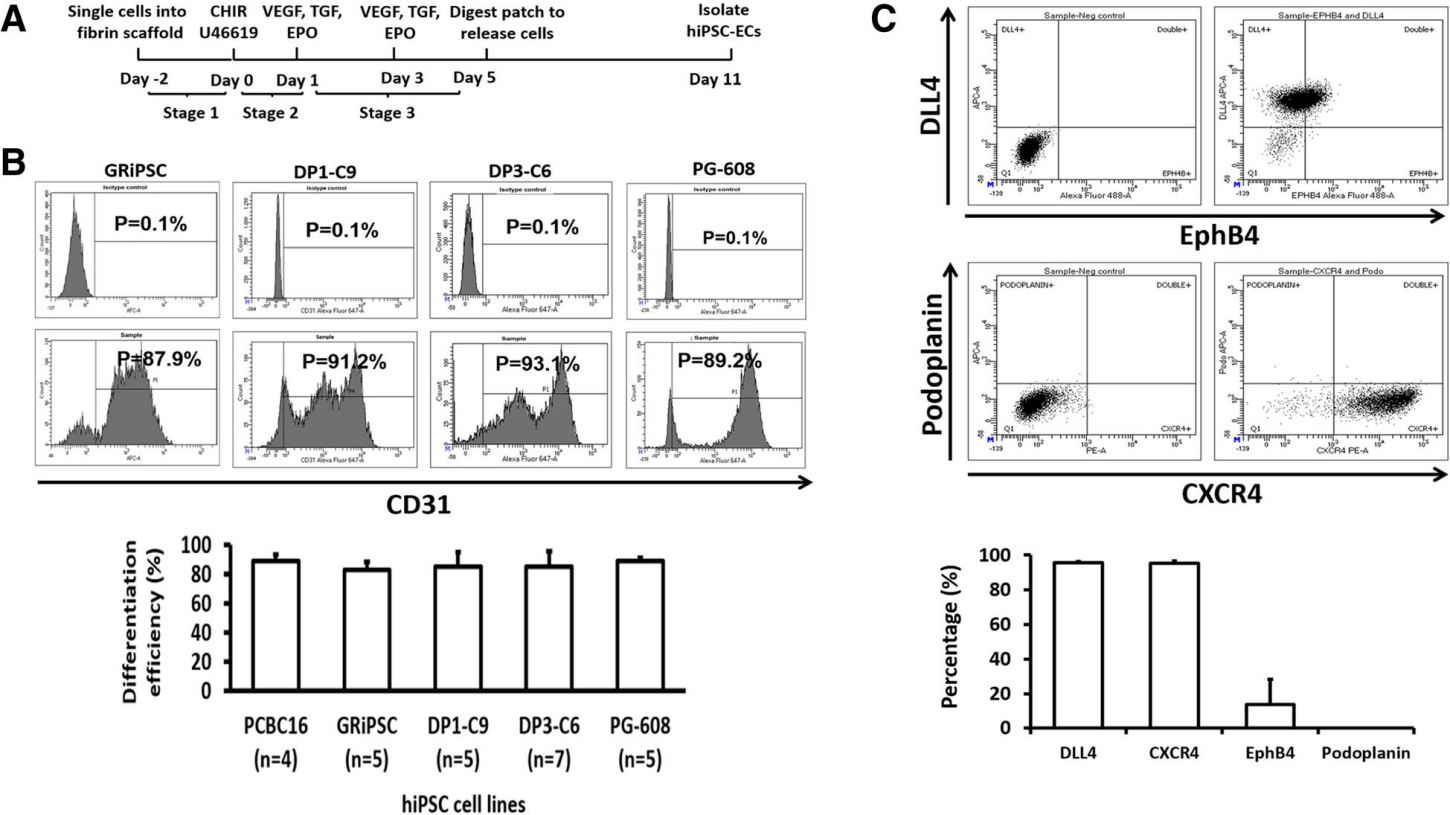
**a** A schematic diagram of the hiPSC-EC differentiation protocol with U46619 treatment in differentiation stage 2. **b** Representative flow cytometry results and hiPSC-EC differentiation efficiency in four hiPSC cell lines: GRiPSC, DP1-C9, DP3-C6, and PG-608. Assessments of CD31 levels were compared with isotype controls. **c** Representative flow cytometry analysis for protein expression of delta-like canonical Notch ligand 4 (DLL4), ephrin B4 (EphB4), podoplanin, and CXCR4 and the percentage of hiPSC-ECs that expressed DLL4, CXCR4, EphB4, and Podoplanin. (*n* = 4)

**Fig. 5:**
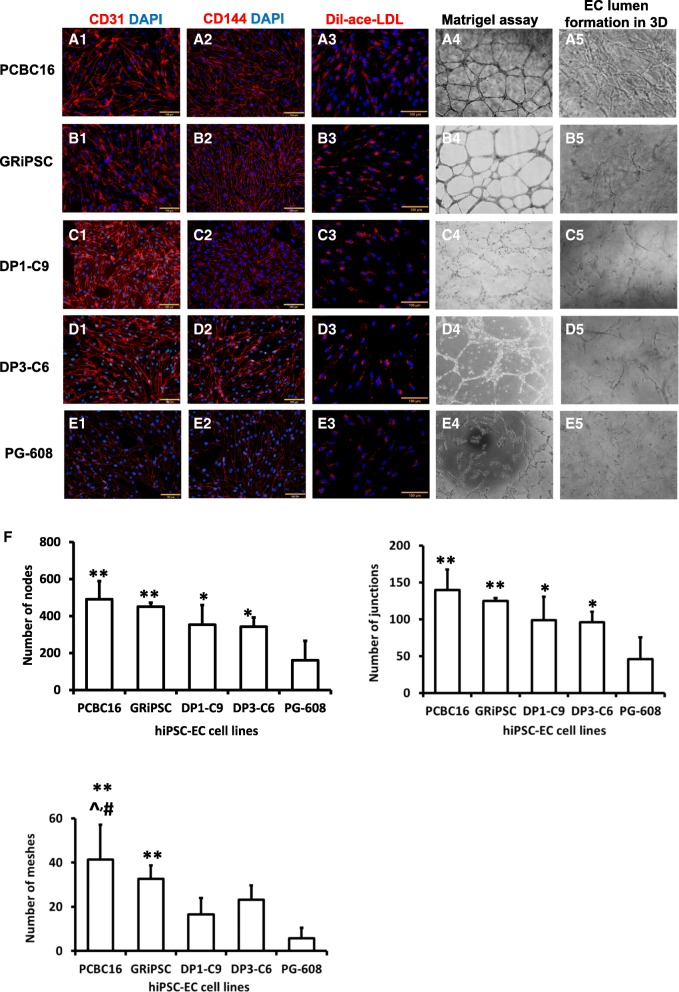
hiPSC-ECs were differentiated from **A** PCBC16, **B** GRiPSC, **C** DP1-C9, **D** DP3-C6, and **E** PG-608 lines and evaluated for the expression of (1) CD31 and (2) CD144, for (3) Dil-ace-LDL uptake, for (4) tube formation on Matrigel, and for (5) EC lumen formation in fibrin-thrombin scaffolds. (Bar = 100 μm. Magnification of tubule formation and EC lumen = × 4). **F** Quantifications of numbers of nodes, junctions, and meshes formed by ECs on Matrigel. (*n* = 5 each for PCBC16, GRiPSC, DP1-C9, and DP3-C6; *n* = 4 for PG-608). (*p < 0.05 and **p < 0.01 vs PG-608. ^*p* < 0.01 vs DP1-C9. ^#^*p* < 0.05 vs DP3-C6)

The dramatically enhanced differentiation efficiency achieved with U-46619 treatment prompted us to modify our protocol by adding U-46619 (1 μM) to the medium during stage 2 (Fig. 4a). The modified protocol was tested in four additional hiPSC lines: GRiPS, which has been well characterized in another study [18]; DP1-C9 and DP3-C6, which were derived from patients with T2DM; and PG-608, which was derived from a patient with Hutchinson-Gilford progeria syndrome; all four lines were reprogrammed from dermal fibroblasts. The differentiation efficiency, as determined via flow cytometry analysis of CD31 expression, exceeded 85% in all lines tested (Fig. 4b). After purification, > 95% of the cells expressed the arteriovenous EC marker CXCR4 [33, 34] and the arterial marker DLL4 [33, 35–37], while ~ 16% expressed the venous marker EphB4 [38, 39], but expression of the lymphatic EC marker podoplanin [40, 41] was undetectable (Fig. 4c). The differentiated cells also co-expressed CD31 and CD144, and functional assessments confirmed that the cells were capable of Dil-ace-LDL uptake and formed tubular structures on Matrigel, as well as EC lumen in fibrin-thrombin scaffolds (Fig. 5A–E). However, the formation of tubular structures and EC lumen was less extensive for ECs differentiated from disease-specific hiPSC lines (particularly PG-608) rather than non-disease hiPSC lines. Quantification showed that ECs derived from PCBC and GRiPSCs had the highest numbers of nodes and junctions (both ~ 3-fold of PG-608), followed by DP1-C9 and DP3-C6 (both ~ 2-fold of PG-608) as compared with PG-608 (Fig. 5F). Furthermore, the numbers of meshes formed by PCBC16 and GRiPSCs were 7- or 5.5-folds of PG-608, while the numbers of meshes formed by DP1-C9 or DP3-C6 were 3- or 4-folds of PG-608.

When the same cell lines were differentiated in monolayers, the differentiation efficiencies were ~ 75% for PCBC16 and GRiPSC cells and 47–63% for the three disease-specific hiPSC lines (Additional file 1: Figure S6). Population-doubling times were also 40–57% shorter in PCBC16-ECs and GRiPSC-ECs (*p* < 0.05), and tended to be shorter in disease-specific hiPSC-ECs (but not significantly), when the cells were differentiated in 3D scaffolds rather than in monolayers (Additional file 1: Figure S7). Thus, the modified 3D differentiation protocol was exceptionally efficient, yielded arteriovenous, but not lymphatic ECs, and may produce cells that are more proliferative than those achieved when the cells are differentiated in monolayers.

### hiPSC-EC transplantation improves perfusion and vascularity in the ischemic limbs of mice

**Fig. 6:**
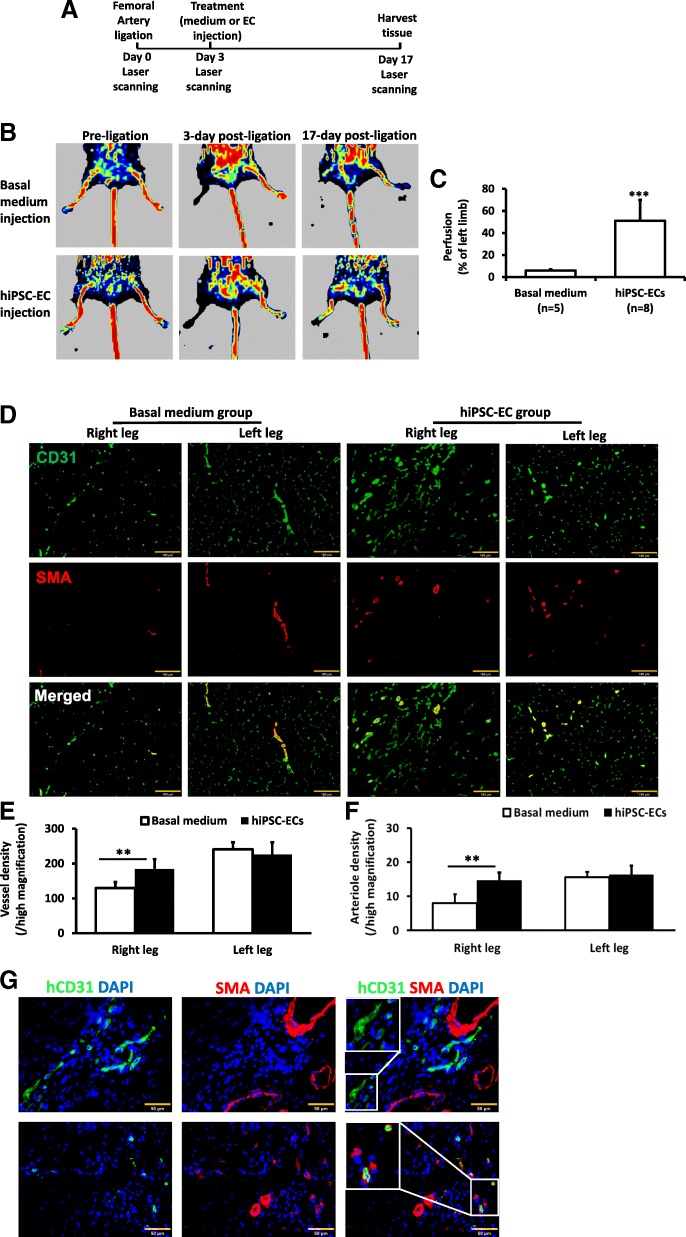
**a** A schematic diagram of the HLI model and treatment. **b** Laser Doppler imaging of mouse limbs before femoral artery ligation, 3 days after femoral artery ligation (i.e., at the time of treatment administration) and 17 days after treatment with basal medium or hiPSC-ECs. Laser Doppler imaging was performed with a PeriScan PIM 3 System with a similar setting. **c** Recovery of right limb perfusion was expressed as a percentage of measurements in the uninjured contralateral limb. **d** Fluorescence staining for CD31 and smooth muscle actin (SMA) in the ischemic limb (right leg) and uninjured limb (left leg) of animals treated with basal medium or hiPSC-ECs after femoral artery ligation. **e** Vessel density and **f** arteriole density in ischemic limbs and uninjured contralateral limbs. **g** Fluorescence staining for human-specific CD31 and SMA in the injured limbs of hiPSC-EC-treated animals (**p < 0.01 and ***p < 0.001; Bar: *D* = 100 μm, *G* = 50 μm)

The hiPSC-ECs produced via our U-46619-enhanced 3D differentiation protocol were also evaluated in a murine HLI model (Fig. 6a). HLI was surgically induced via permanent ligation of the right femoral artery. Three days later, animals in the hiPSC-EC group (*n* = 8) were treated with injections of basal medium containing 1.5 × 10^6^ hiPSC-ECs, and animals in the BM group (*n* = 9) were treated with an equivalent volume of cell-free basal medium. The hiPSC-ECs had been differentiated from PCBC16 cells, and injections were administered directly into the ischemic limb muscle. Perfusion was evaluated in both the ischemic and non-ischemic contralateral limbs immediately before HLI induction, at the time of treatment administration and 2 weeks after treatment via laser Doppler imaging (Fig. 6b). Measurements in the ischemic limb were normalized to measurements in the non-ischemic contralateral limb.

No evidence of perfusion was observable in images obtained at the time of cell administration, and the ischemic limbs of animals in the BM group were lost in four (out of nine) mice by week 2. However, all limbs were retained by animals in the hiPSC-EC group, and perfusion in their ischemic limbs had recovered to approximately 50% of measurements in their non-ischemic limbs, which was significantly greater than the extent of recovery observed in the five BM-treated mice that had not lost their ischemic limbs (6%, *p* < 0.001) (Fig. 6c). Furthermore, assessments in sections stained for CD31 and SMA (Fig. 6d) indicated that measurements of total vessel density and arterial density in the ischemic limbs of animals in the hiPSC-EC group were ~ 80% (Fig. 6e) and ~ 90% (Fig. 6f), respectively, of measurements in their non-ischemic limbs and significantly higher than measurements in the ischemic limbs of BM-treated mice. Tissue sections stained for SMA and the human-specific isoform of hCD31 indicated that the transplanted hiPSC-ECs were present both in smooth muscle-containing vessels and in vessels that lacked smooth muscle (Fig. 6g), suggesting that transplanted hiPSC-ECs can contribute to capillary and arteriole formation. Collectively, these observations suggest that transplanted hiPSC-ECs can restore perfusion in the ischemic limb muscles of mice by promoting neovascularization.

## Discussion

In a previous report, we demonstrated that hiPSCs can be differentiated into ECs with reasonable efficiency (~ 45%) by culturing the cells in a 3D fibrin scaffold, rather than as a 2D layer [18]. The protocol consisted of three stages: in stage 1, the hiPSCs were seeded into the scaffold and maintained under standard conditions; then, in stage 2, the cells were directed toward the mesodermal lineage by culturing them with Activin-A and BMP4, and the EC phenotype was induced in stage 3 by exposing the cells to VEGF, TGF, and EPO. For the experiments reported here, we used the GSK-3α/β inhibitor CHIR rather than Activin-A and BMP4 in stage 2, which modestly improved the efficiency of our protocol (to ~ 58%). Then, we identified two signaling pathways, p38MAPK and MEK/ERK, that contributed to hiPSC-EC differentiation. We upregulated the activities of those pathways by adding the prostaglandin H2 analog U-46619 to the medium in stage 2. The differentiation efficiency achieved with this enhanced protocol was as high as 89% when evaluated in an established hiPSC line and between 83 and 90% in all five hiPSC lines tested. Three cell lines were reprogrammed from the cells of patients with diseases or disease symptoms that are associated with endothelial dysfunction: T2DM (two lines) [23] and Hutchinson-Gilford progeria syndrome (one line) [24, 25]. Analysis of the expression of markers for arterial (DLL4 and CXCR4) [33–37], venous (EphB4 and CXCR4) [33, 34, 38, 39], and lymphatic (podoplanin) [40, 41] ECs indicated that the population of differentiated cells contained only arteriovenous ECs. hiPSC-ECs formed tubular structures and EC lumens in vitro and restore perfusion and improve vascularity in a murine HLI model after transplantation in vivo.

Although both ERK1/2 and p38MAPK signaling participate in the differentiation of hiPSC-ECs, their involvement during our protocol appears to be of varying importance. Treatment with the ERK1/2 inhibitor *SCH* led to declines in Brachyury expression when the inhibitor was added during stage 2 and in the expression of Etv2, a master regulator of EC development [42, 43], when it was added in stage 3. Thus, ERK1/2 activity is required for both the mesodermal specification of hiPSCs [44] and for inducing the terminal EC phenotype. Some evidence suggests that p38MAPK activity is also required for Etv2 gene expression during endothelial differentiation [19]. However, hiPSC-EC differentiation was only impaired, not blocked, by treatment with the p38MAPK inhibitor Losma, and Etv2 expression levels only declined when Losma was added in stage 2, but not when it was added in stage 3. Losma treatment also reduced, while SCH treatment blocked, hiPSC-EC differentiation in the presence of U-46619, which activated p38MAPK and ERK1/2 [35, 36] and increased the magnitude and duration of the expression of endothelial transcription factors such as Etv2, Gata-2, and Tal-1. Thus, although our observations confirm that both p38MAPK and ERK1/2 activities contribute to hiPSC-EC differentiation, ERK1/2 appears to be indispensable, while p38MAPK likely serves in an auxiliary role.

Our hiPSC-EC differentiation protocol produced cells with shorter population-doubling times, indicating that the 3D environment enhanced cell growth and proliferation [45]. Furthermore, the efficiency of our differentiation protocol exceeded 85% when tested with hiPSCs that had been reprogrammed from the cells of patients whose disease or disease symptoms have been linked to endothelial dysfunction. These observations are notable because the biological activity of disease-specific stem/progenitor cells is often impaired [46–48]. The protocol may also overcome epigenetic factors that the hiPSCs retain from their tissues of origin (i.e., epigenetic memory) [49] and, consequently, could improve hiPSC-EC differentiation in hiPSC lines that have been reprogrammed from non-endothelial cells.

## Conclusion

We have developed an enhanced 3D protocol for differentiating hiPSCs into ECs that uses the prostaglandin H2 analog U-46619 to upregulate p38MAPK and ERK1/2 activity. The protocol produced populations of arteriovenous ECs that were up to 89% pure, formed tubular structures and EC lumens in vitro, and restored perfusion and improved vascularity in a murine HLI model after transplantation in vivo. Collectively, these observations have important implications for the use of ECs in tissue engineering or as an in vitro platform for drug testing and disease modeling.

## Additional file

Additional file 1: **Figure S1.** Western blot analysis for protein expression of phosphorylated p38MAPK (p-p38MAPK), p38MAPK, phosphorylated ERK1/2 (p-ERK1/2), ERK1/2, and internal control GAPDH in differentiating hiPSCs treated with CHIR, or Losma+CHIR, or SCH+CHIR at stage 2. **Figure S2.** (A) Brachyury gene expression level in presence of SCH and/or Losma at stage 2. Gene expression levels of Etv2 (B), Gata-2 (C), Tal-1 (D), CD34 (E), and CD31 (F) as a function of differentiation time when SCH and/or Losma was supplemented in differentiation stage 2. Gene expression levels of Etv2 (G), Gata-2 (H), Tal-1 (I), CD34 (J), and CD31 (K) as a function of differentiation time when SCH and/or Losma was supplemented in differentiation stage 3. **Figure S3.** (A) Typical flow cytometry result of hiPSC-EC differentiation efficiency when 5 μM U46619 was supplemented in differentiation stages 2. The proportion of cells expressed CD31 were compared with respective isotype controls. (B) Mean differentiation efficiency of hiPSC-ECs when 5 μM U46619 was supplemented in differentiation stage 2 or 3. **Figure S4.** Western blot analysis for protein expression of p-p38MAPK, p38MAPK, p-ERK1/2, ERK1/2, and internal control GAPDH in differentiating hiPSCs treated with U46619, or Losma+U46619, or SCH+U46619, or Losma+SCH+U46619. **Figure S5.** (A) Brachyury gene expression level in presence of U46619, or SCH and/or Losma at stage 2. Gene expression levels of Etv2 (B), Gata-2 (C), Tal-1 (D), CD34 (E), and CD31 (F) as a function of differentiation time when U46619, or SCH and/or Losma was supplemented in differentiation stage 2. Gene expression levels of Etv2 (G), Gata-2 (H), Tal-1 (I), CD34 (J), and CD31 (K) as a function of differentiation time when U46619, or SCH and/or Losma was supplemented in differentiation stage 3. **Figure S6.** hiPSC-EC differentiation efficiencies when hiPSC were differentiated in monolayer. Figure S7. Cell doubling time of ECs differentiated in 3D or monolayers. (PPT 2185 kb)

## Abbreviations

3D: 3-Dimensional
APC: Allophycocyanin
BM: Basal medium
BMP-4: Bone morphogenetic protein 4
CHIR: CHIR99021
CXCR4: C-X-C chemokine receptor type 4
DAPI: 4′,6-Diamidino-2-phenylindole
Dil-ace-LDL: Dil-conjugated acetylated low-density lipoprotein
DLL4: Delta-like 4
E8BAC: E8 medium supplemented 5 ng/mL BMP4, 25 ng/mL Activin-A, and 1 μM CHIR
EBM: Endothelial basal medium
EC: Endothelial cell
EDTA: Ethylenediaminetetraacetic acid
EGM: Endothelial growth medium
EphB4: Ephrin B4
EPO: Erythropoietin
ERK1/2: Extracellular signal-regulated protein kinases 1 and 2
FACS: Fluorescence-activated cell sorting
FBS: Fetal bovine serum
FGF2: Fibroblast growth factor 2
FITC: Fluorescein
FVIR: DMEM/F12 medium supplemented with 64 ng/mL l-ascorbic acid-2-phosphate-magnesium, 14 ng/mL sodium selenium, 534 μg/mL NaHCO3, 10.7 μg/mL transferrin, 20 μg/mL insulin, 100 ng/mL fibroblast growth factor 2 (FGF2), 50 ng/mL VEGF, 10 μM SB, and 5 μM RESV
GAPDH: Glyceraldehyde 3-phosphate dehydrogenase
hCD31: Specific anti-human CD31 antibody.
hiPSC-ECs: Endothelial cells differentiated from human induced-pluripotent stem cells
hiPSCs: Human-induced pluripotent stem cells
HLI: Hind limb ischemia
L690: L690330
Losma: Losmapimod
p38MAPK: p38 mitogen-activated protein kinase
PBS: Phosphate-buffered saline
PE: Phycoerythrin
qRT-PCR: Quantitative reverse transcription polymerase chain reaction
RESV: Resveratrol
SB: SB-431542
SCH: SCH772984
SD: Standard deviation
SMA: Smooth muscle actin
T2DM: Type 2 diabetes mellitus
TGFβ1: Transforming growth factor β1
VEGF: Vascular endothelial growth factor 165
