# Supplementary material for: The prostaglandin H2 analog U-46619 improves the differentiation efficiency of human induced pluripotent stem cells into endothelial cells by activating both p38MAPK and ERK1/2 signaling pathways
Source: Stem Cell Res Ther. 2018 Nov 15;9:313. doi: 10.1186/s13287-018-1061-4 (PMC6238266; doi:10.1186/s13287-018-1061-4)
Supplement: Supplementary file 1 — Figure S1. Western blot analysis for protein expression of phosphorylated p38MAPK (p-p38MAPK), p38MAPK, phosphorylated ERK1/2 (p-ERK1/2), ERK1/2, and internal control GAPDH in differentiating hiPSCs treated with CHIR, or Losma+CHIR, or SCH+CHIR at stage 2. Figure S2. (A) Brachyury gene expression level in presence of SCH and/or Losma at stage 2. Gene expression levels of Etv2 (B), Gata-2 (C), Tal-1 (D), CD34 (E), and CD31 (F) as a function of differentiation time when SCH and/or Losma was supplemented in differentiation stage 2. Gene expression levels of Etv2 (G), Gata-2 (H), Tal-1 (I), CD34 (J), and CD31 (K) as a function of differentiation time when SCH and/or Losma was supplemented in differentiation stage 3. Figure S3. (A) Typical flow cytometry result of hiPSC-EC differentiation efficiency when 5 μM U46619 was supplemented in differentiation stages 2. The proportion of cells expressed CD31 were compared with respective isotype controls. (B) Mean differentiation efficiency of hiPSC-ECs when 5 μM U46619 was supplemented in differentiation stage 2 or 3. Figure S4. Western blot analysis for protein expression of p-p38MAPK, p38MAPK, p-ERK1/2, ERK1/2, and internal control GAPDH in differentiating hiPSCs treated with U46619, or Losma+U46619, or SCH+U46619, or Losma+SCH+U46619. Figure S5. (A) Brachyury gene expression level in presence of U46619, or SCH and/or Losma at stage 2. Gene expression levels of Etv2 (B), Gata-2 (C), Tal-1 (D), CD34 (E), and CD31 (F) as a function of differentiation time when U46619, or SCH and/or Losma was supplemented in differentiation stage 2. Gene expression levels of Etv2 (G), Gata-2 (H), Tal-1 (I), CD34 (J), and CD31 (K) as a function of differentiation time when U46619, or SCH and/or Losma was supplemented in differentiation stage 3. Figure S6. hiPSC-EC differentiation efficiencies when hiPSC were differentiated in monolayer. Figure S7. Cell doubling time of ECs differentiated in 3D or monolayers. (PPT 2185 kb) [file 13287_2018_1061_MOESM1_ESM.ppt]

## Slide 1
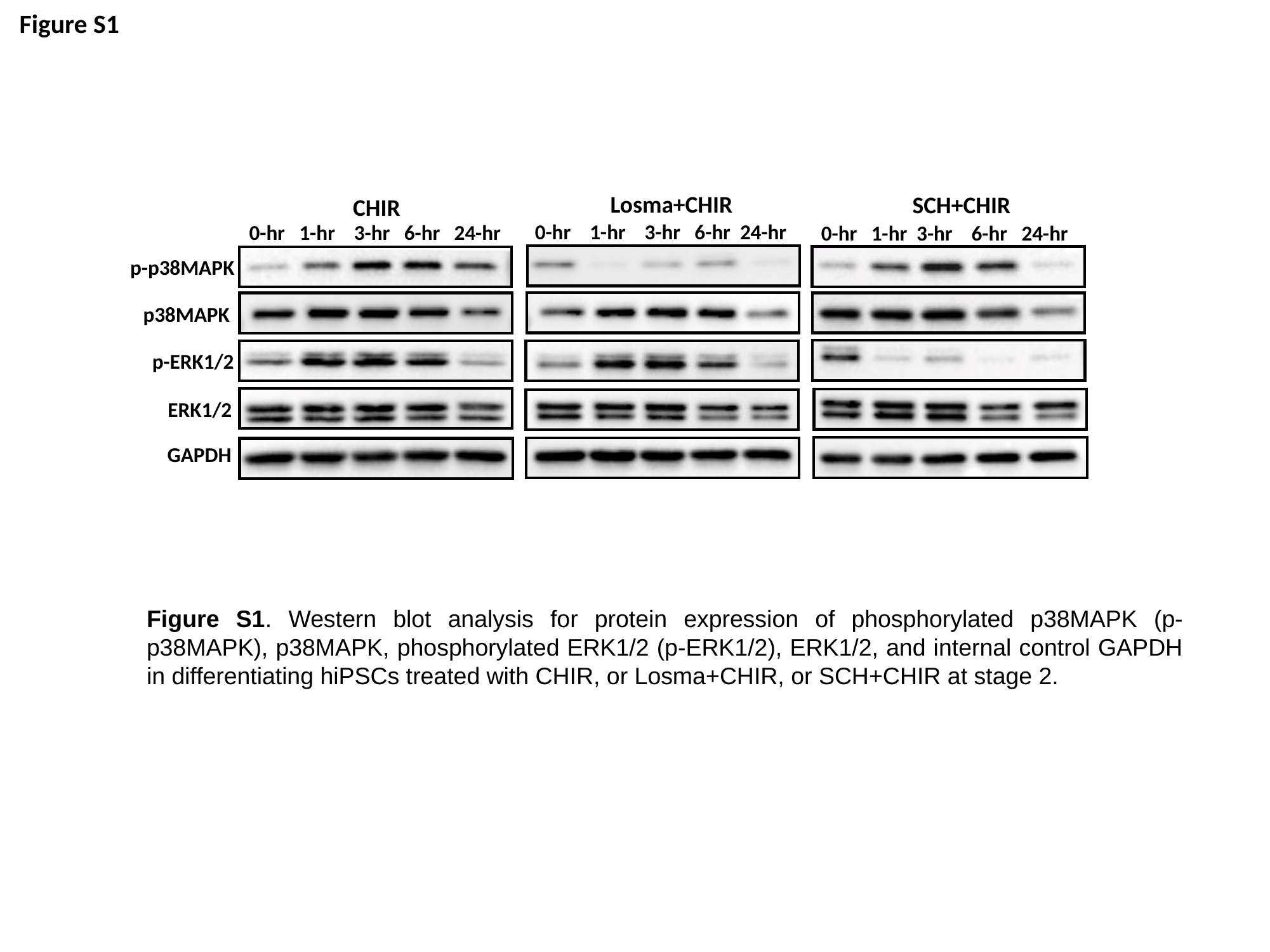

Figure S1
Losma+CHIR
0-hr 1-hr 3-hr 6-hr 24-hr
SCH+CHIR
0-hr 1-hr 3-hr 6-hr 24-hr
CHIR
0-hr 1-hr 3-hr 6-hr 24-hr
p-p38MAPK
p38MAPK
p-ERK1/2
ERK1/2
GAPDH
Figure S1. Western blot analysis for protein expression of phosphorylated p38MAPK (p-p38MAPK), p38MAPK, phosphorylated ERK1/2 (p-ERK1/2), ERK1/2, and internal control GAPDH in differentiating hiPSCs treated with CHIR, or Losma+CHIR, or SCH+CHIR at stage 2.

## Slide 2
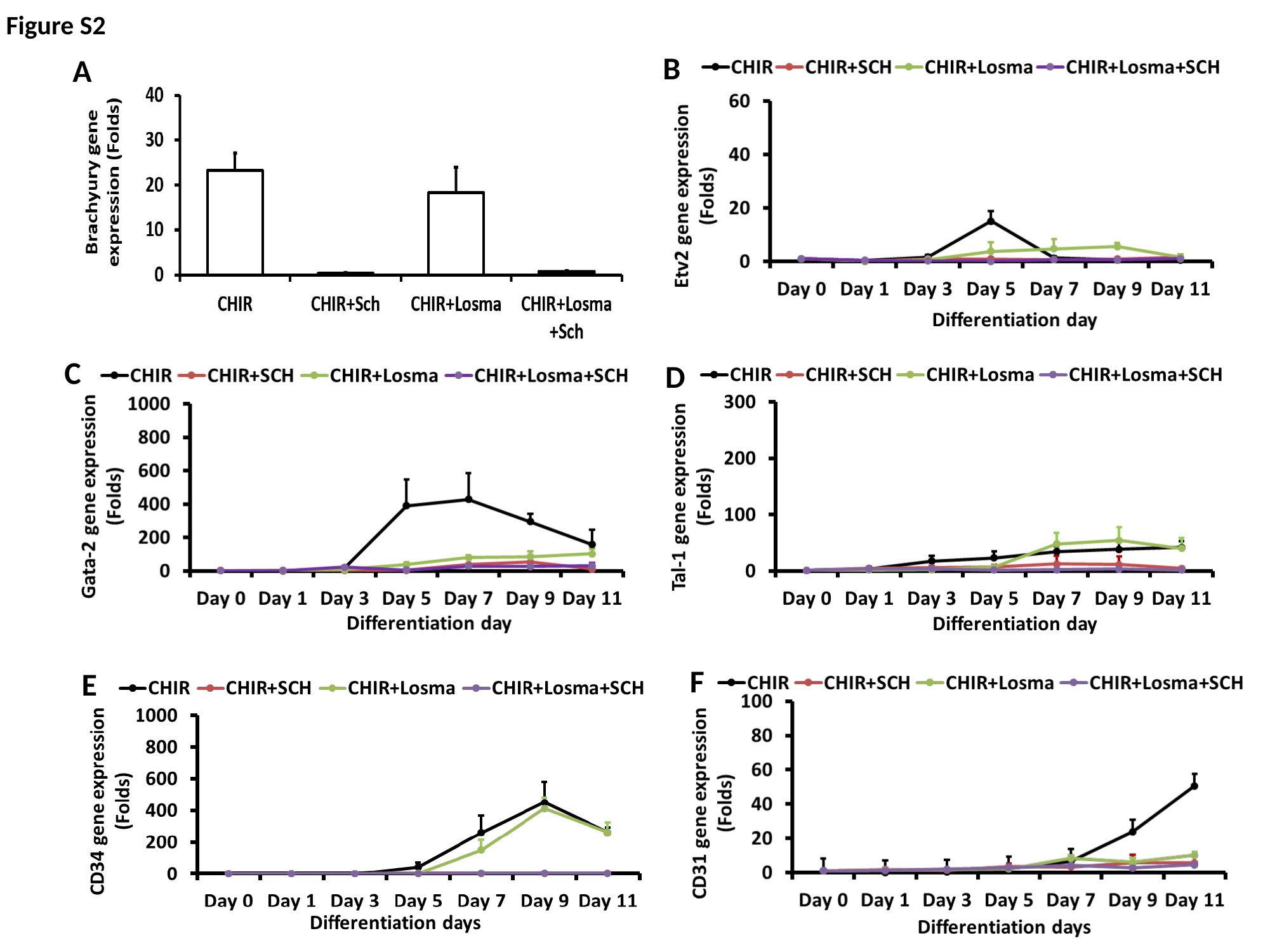

Figure S2
B
A
C
D
F
E

## Slide 3
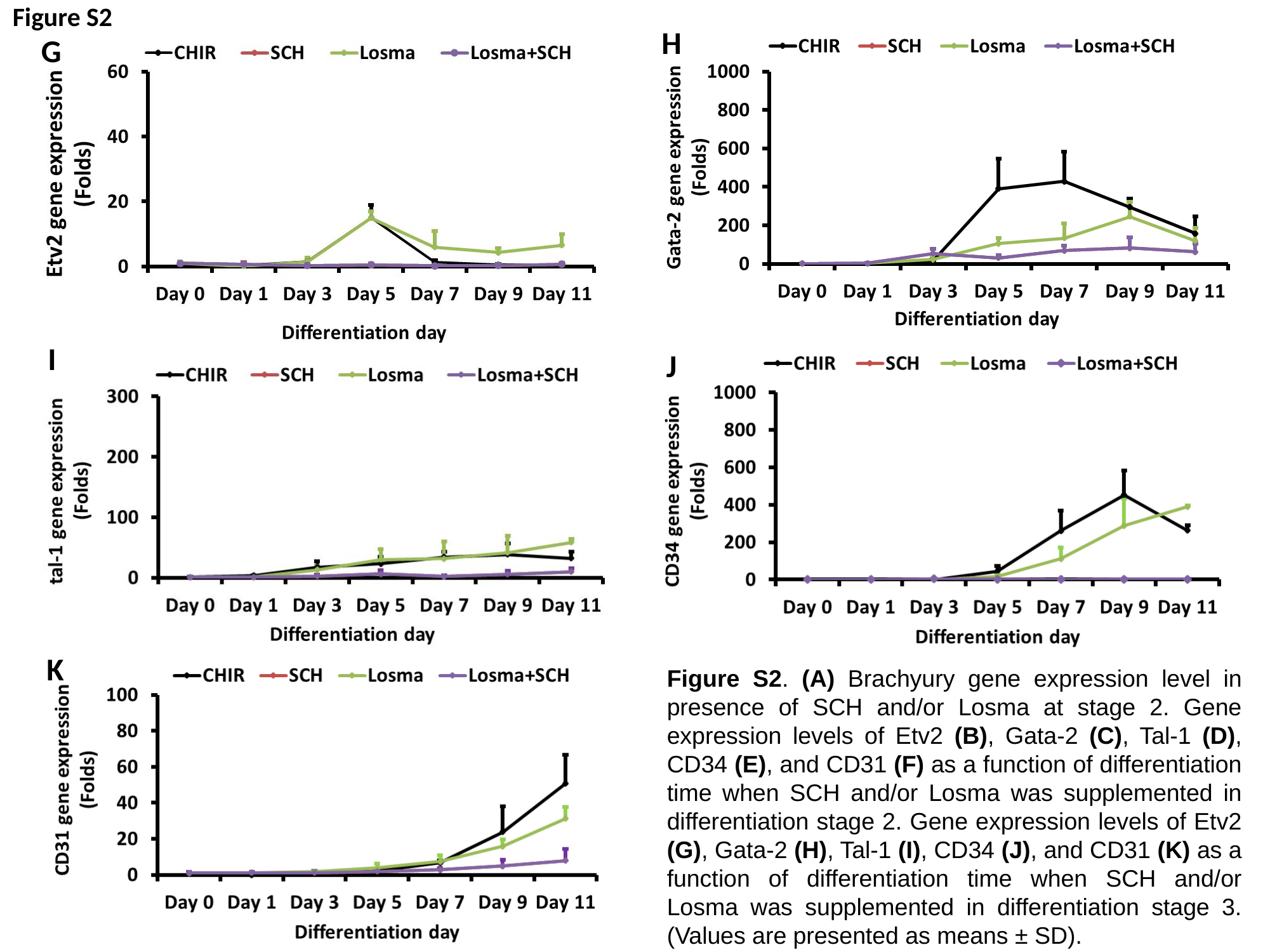

Figure S2
H
G
I
J
K
Figure S2. (A) Brachyury gene expression level in presence of SCH and/or Losma at stage 2. Gene expression levels of Etv2 (B), Gata-2 (C), Tal-1 (D), CD34 (E), and CD31 (F) as a function of differentiation time when SCH and/or Losma was supplemented in differentiation stage 2. Gene expression levels of Etv2 (G), Gata-2 (H), Tal-1 (I), CD34 (J), and CD31 (K) as a function of differentiation time when SCH and/or Losma was supplemented in differentiation stage 3. (Values are presented as means ± SD).

## Slide 4
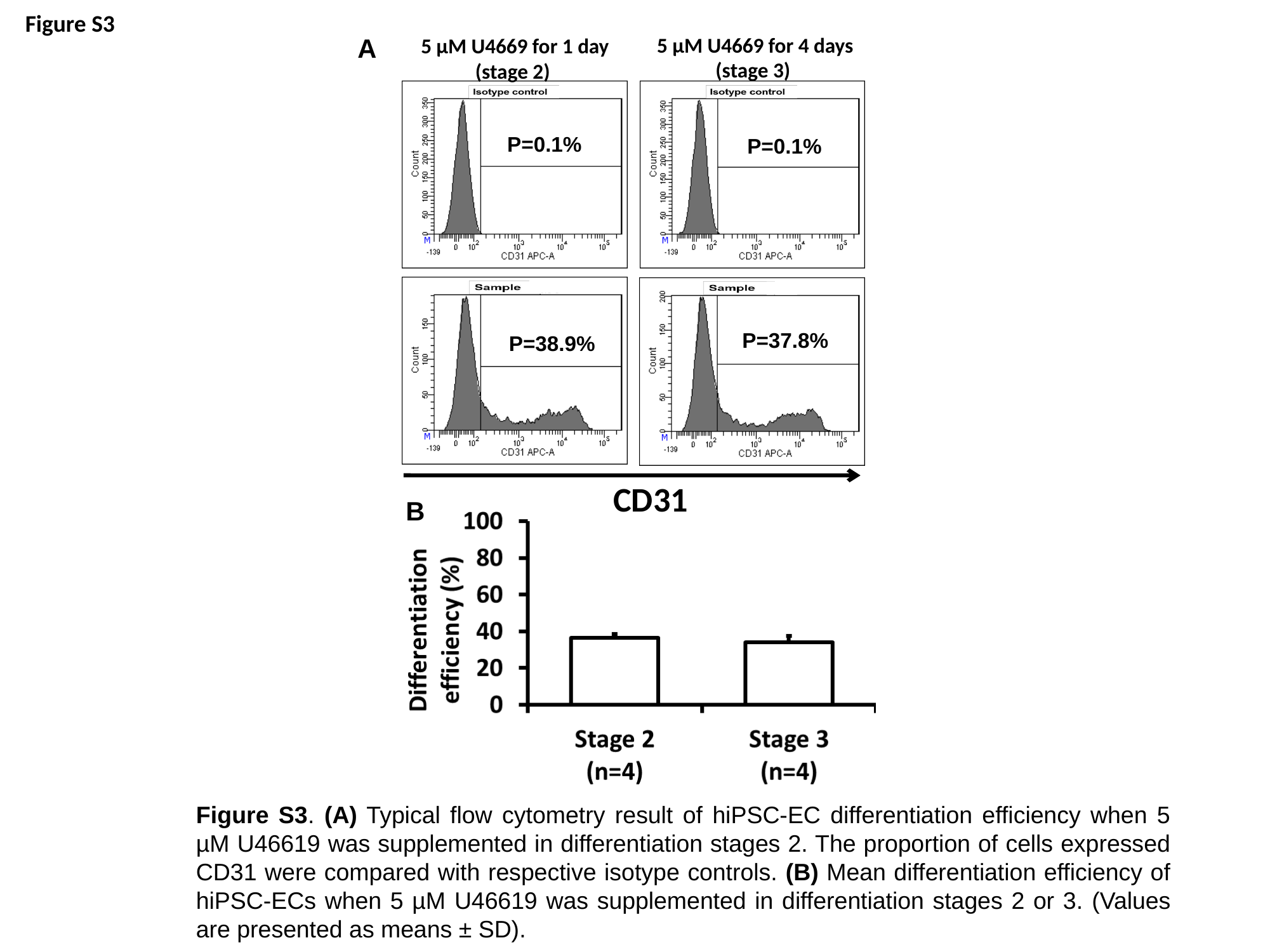

Figure S3
A
5 µM U4669 for 4 days
(stage 3)
5 µM U4669 for 1 day
(stage 2)
P=0.1%
P=0.1%
P=38.9%
P=37.8%
CD31
B
Figure S3. (A) Typical flow cytometry result of hiPSC-EC differentiation efficiency when 5 µM U46619 was supplemented in differentiation stages 2. The proportion of cells expressed CD31 were compared with respective isotype controls. (B) Mean differentiation efficiency of hiPSC-ECs when 5 µM U46619 was supplemented in differentiation stages 2 or 3. (Values are presented as means ± SD).

## Slide 5
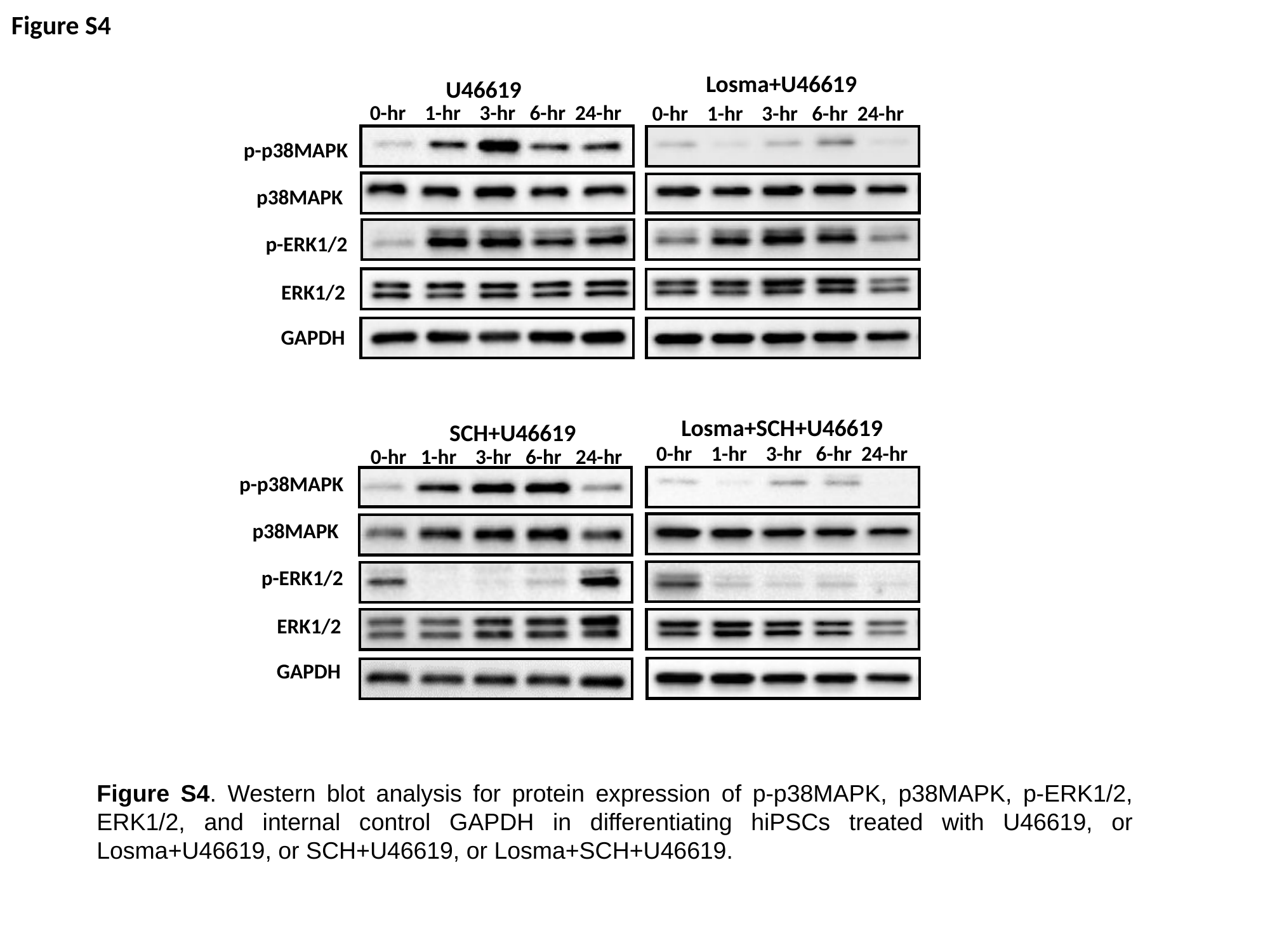

Figure S4
Losma+U46619
 U46619
0-hr 1-hr 3-hr 6-hr 24-hr
0-hr 1-hr 3-hr 6-hr 24-hr
p-p38MAPK
p38MAPK
p-ERK1/2
ERK1/2
GAPDH
Losma+SCH+U46619
SCH+U46619
0-hr 1-hr 3-hr 6-hr 24-hr
0-hr 1-hr 3-hr 6-hr 24-hr
p-p38MAPK
p38MAPK
p-ERK1/2
ERK1/2
GAPDH
Figure S4. Western blot analysis for protein expression of p-p38MAPK, p38MAPK, p-ERK1/2, ERK1/2, and internal control GAPDH in differentiating hiPSCs treated with U46619, or Losma+U46619, or SCH+U46619, or Losma+SCH+U46619.

## Slide 6
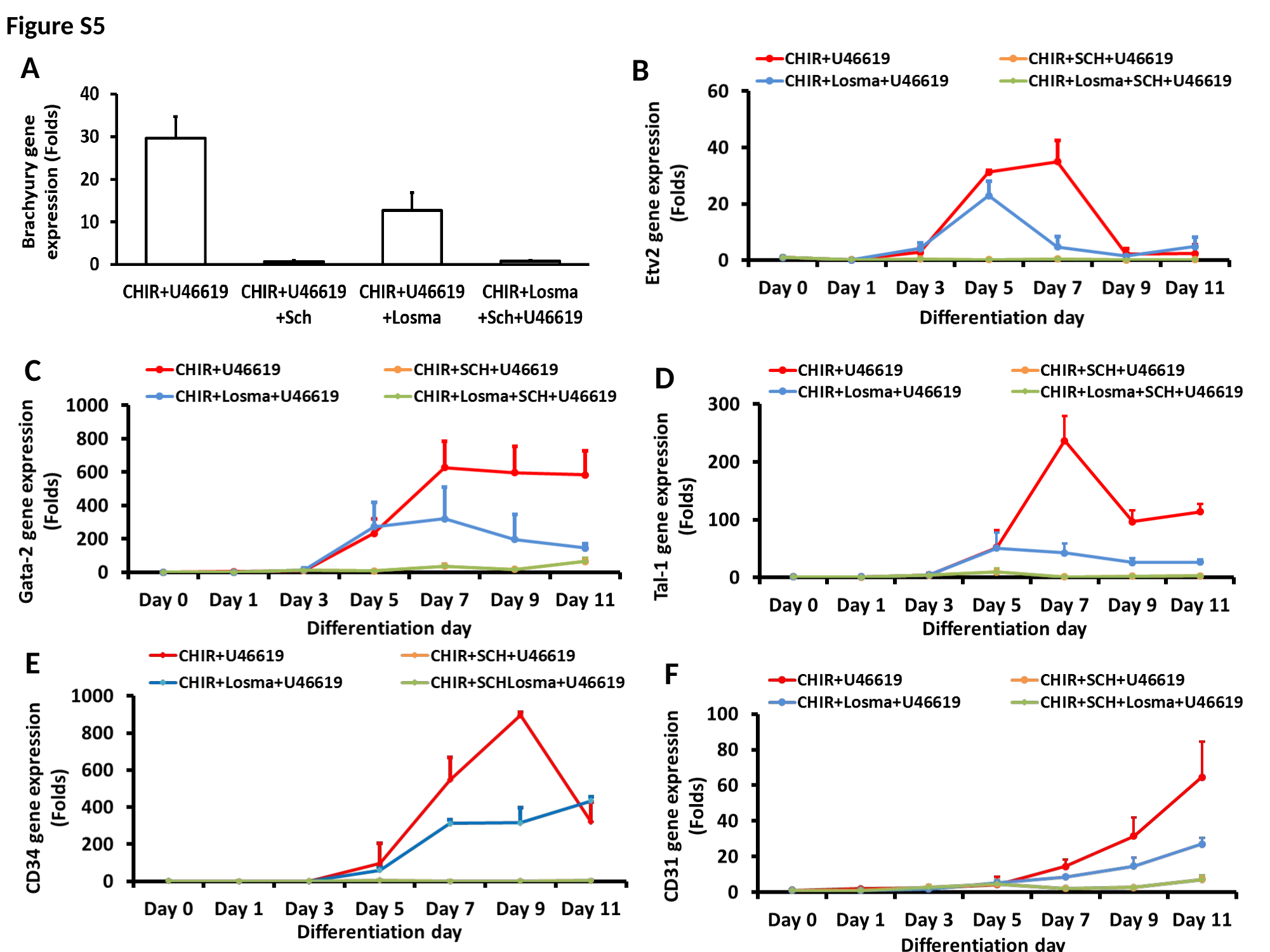

Figure S5
A
B
C
D
E
F

## Slide 7
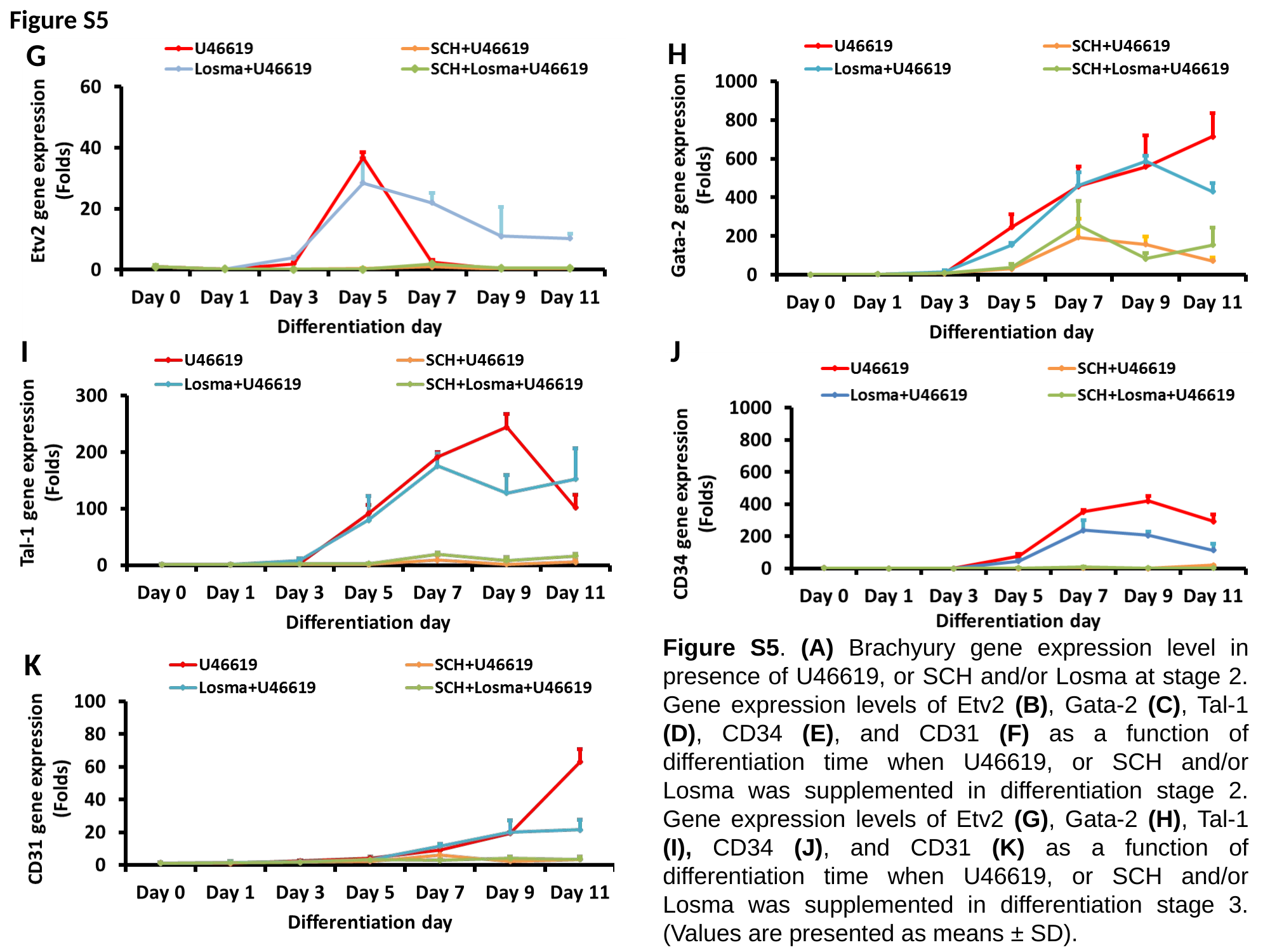

Figure S5
H
G
I
J
Figure S5. (A) Brachyury gene expression level in presence of U46619, or SCH and/or Losma at stage 2. Gene expression levels of Etv2 (B), Gata-2 (C), Tal-1 (D), CD34 (E), and CD31 (F) as a function of differentiation time when U46619, or SCH and/or Losma was supplemented in differentiation stage 2. Gene expression levels of Etv2 (G), Gata-2 (H), Tal-1 (I), CD34 (J), and CD31 (K) as a function of differentiation time when U46619, or SCH and/or Losma was supplemented in differentiation stage 3. (Values are presented as means ± SD).
K

## Slide 8
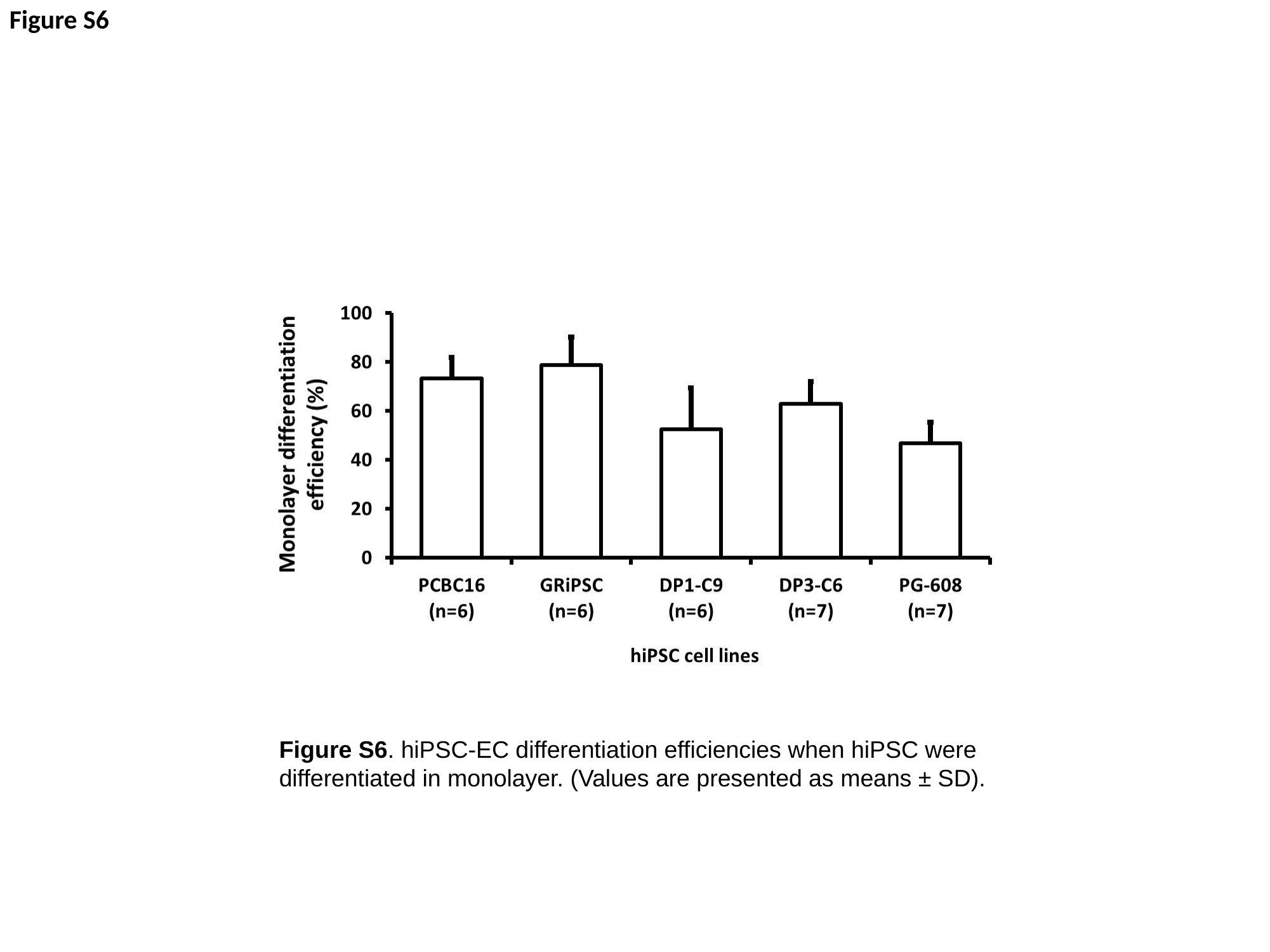

Figure S6
Figure S6. hiPSC-EC differentiation efficiencies when hiPSC were differentiated in monolayer. (Values are presented as means ± SD).

## Slide 9
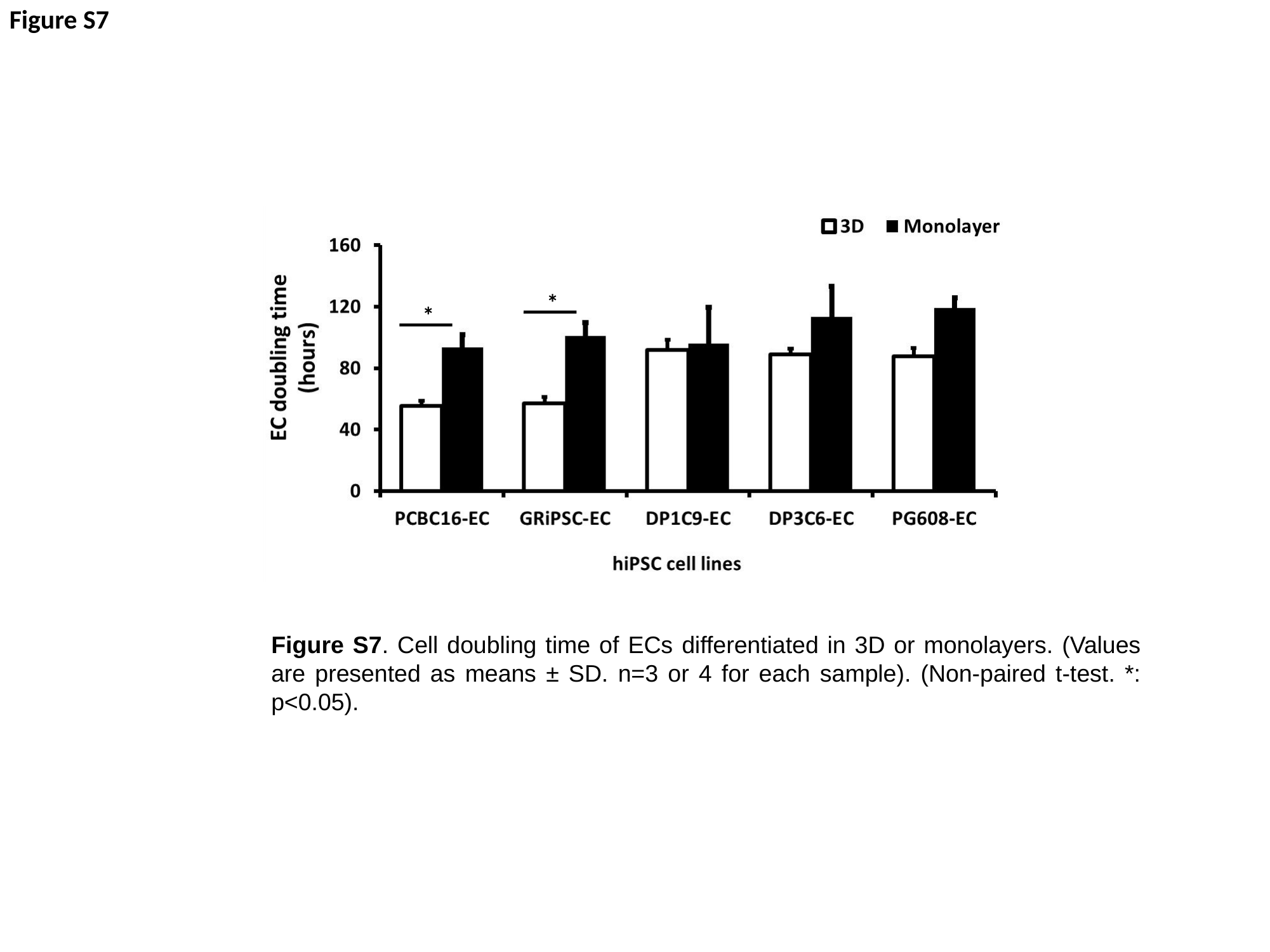

Figure S7
*
*
Figure S7. Cell doubling time of ECs differentiated in 3D or monolayers. (Values are presented as means ± SD. n=3 or 4 for each sample). (Non-paired t-test. *: p<0.05).
